# Long non-coding RNA H19 as a prognostic biomarker for oral squamous cell carcinoma

**DOI:** 10.3389/fmed.2024.1456963

**Published:** 2024-11-26

**Authors:** Kiran Kumar, Kaveri Hallikeri, Ajay Kumar Oli, Kiran Radder, Apoorva Jain, A. S. Shilpasree, Raghu Dhanapal, Abedelmalek Kalefh Tabnjh, Siddharthan Selvaraj

**Affiliations:** ^1^Department of Oral and Maxillofacial Pathology and Microbiology, SDM College of Dental Sciences and Hospital, Shri Dharmasthala Manjunatheshwara University, Dharwad, Karnataka, India; ^2^Department of Biomedical Science, SDM Research Institute for Biomedical Sciences, Shri Dharmasthala Manjunatheshwara University, Dharwad, Karnataka, India; ^3^Craniofacial Unit, SDM College of Medical Sciences and Hospital, Shri Dharmasthala Manjunatheshwara University, Dharwad, Karnataka, India; ^4^Department of Biochemistry, SDM College of Medical Sciences and Hospital, Shri Dharmasthala Manjunatheshwara University, Dharwad, Karnataka, India; ^5^Department of Oral and Maxillofacial Pathology and Microbiology, RVS Dental College and Hospital, Coimbatore, India; ^6^Department of Cariology, Institute of Odontology, The Sahlgrenska Academy, University of Gothenburg, Gothenburg, Sweden; ^7^Department of Applied Dental Sciences, Faculty of Applied Medical Sciences, Jordan University of Science and Technology, Irbid, Jordan; ^8^Department of Public Health Dentistry, Saveetha Dental College and Hospitals, Saveetha Institute of Medical and Technical Sciences, Saveetha University, Chennai, Tamil Nadu, India; ^9^Department of Community Oral Health and Clinical Prevention, Faculty of Dentistry, Universiti Malaya, Kuala Lumpur, Malaysia; ^10^Department of Dental Research Cell, Dr. D. Y. Patil Dental College & Hospital, Dr. D. Y. Patil Vidyapeeth, Pune, India

**Keywords:** oral squamous cell carcinomas, long non-coding RNA, reverse transcriptase PCR, H19 expression, loco-regional recurrence, overall survival

## Abstract

**Background:**

H19, a 2.3 kb lncRNA, has been linked to tumor metastasis and progression, but its significance in oral squamous cell carcinoma (OSCC) remains unclear. H19 was initially thought to have a tumor-suppressive function, but recent studies have shown that it possesses both tumor-promoting and suppressive functions. The variation in H19 expression may be due to the influence of tobacco or low basal expression levels. However, there are limited studies available on the association between H19 and its role in the prognosis of OSCC.

**Objective:**

The present study analyzes the expression of H19 correlated with clinicopathological parameters, tobacco habit, loco-regional recurrence, and overall survival.

**Methods:**

A longitudinal study was undertaken using 96 formalin-fixed paraffin-embedded (FFPE) OSCC tissues and 30 FFPE adjacent normal mucosa (NM) tissues from patients who had surgery between 2015 and 2018. The tissues were subjected to quantitative reverse-transcription PCR (qRT-PCR) to determine H19 expression. The differential expression levels of H19 in OSCC were compared to clinicopathological variables and risk habits using the t-test and ANOVA. H19 expression correlated overall survival was analyzed by drawing the Kaplan–Meier curve followed by the log-rank test. A multivariate Cox proportional hazards regression analysis was performed to determine the ability of H19 to independently predict loco-regional recurrence and overall survival for OSCC.

**Result:**

H19 was significantly underexpressed in OSCC compared to NM in both the study cases and the TCGA OSCC database. The lower expression of H19 was significantly associated with the tobacco smoking habit and was not associated with any clinical or pathological features. Multivariate Cox’s proportional hazards regression analysis indicated that low H19 expression and positive lymph node metastasis were independent predictors of overall survival for OSCC. Higher age, higher TNM staging, and low H19 expression were independent predictors of loco-regional recurrence.

**Conclusion:**

The findings in the present study indicate that H19 is a novel prognostic marker and may provide a therapeutic strategy for the targeted treatment of OSCC, and tobacco may play a role in the expression of H19.

## 1 Introduction

Head and neck squamous cell carcinomas (HNSCCs) are the seventh most common cancer in the world involving the oral cavity, pharynx, larynx, paranasal cavity, nasal cavity, and salivary glands (1). As per Global Cancer Observatory (GLOBOCON) 2020 estimates, oral squamous cell carcinoma (OSCC) arising from the oral cavity and lips is the most common type of malignancy among HNSCCs, mainly associated with chewing of areca nuts (betel quid), with or without tobacco (2). In India, approximately 77,000 new cases and 52,000 deaths due to OSCC are reported annually, which is approximately one-fourth of global incidences (3). As compared to the developed countries, 70% of the OSCC cases are reported in the advanced stages (American Joint Committee on Cancer, stages III–IV). Because of detection in the late phase, the chances for cure are very low, almost negative, leaving 5-year survival rates of approximately 20% only (4). Hence, discovering suitable predictive biomarkers is key to preventing relapse and improving the survival of patients with OSCC.

Improvements in transcriptomics have increased our understanding of the molecular processes underlying cancer biology (5). The RNA-seq methods have recently been used by the Encyclopedia of DNA Elements (ENCODE) project to identify and describe novel and annotated RNA transcripts that do not code for any protein known as non-coding RNAs (ncRNAs) (6). Among them, long non-coding RNA (lncRNA) is a novel class of ncRNAs comprised of over 200 nucleotides, which have been implicated in the pathogenesis of many types of cancer, including OSCCs, and may serve as potential therapeutic targets (7).

H19 is a 2.3 kb lncRNA molecule first reported as upregulated in bladder carcinoma and has been recognized as a predictive marker for early recurrence (5, 8). Previous studies have reported aberrant expression in various cancers, such as pancreatic cancer (9), breast cancer (10), colorectal carcinoma (11), and hepatocellular carcinoma (12). This expression correlates with cancer progression, metastasis, and poor prognosis, suggesting that H19 may be used as a diagnostic and prognostic biomarker.

H19 was initially thought to have tumor-suppressive function because of its ability to inhibit tumorigenesis, but recent studies have now shown that H19 possesses both tumor promoter and suppressive functions (13).

There is heterogeneity in the results of studies analyzing H19 expression in OSCCs. Studies have reported significant overexpression of H19 in OSCCs mainly involving the tongue compared to adjacent normal mucosa and was correlated with poor prognosis (8, 14, 15). Whereas, two Indian studies on mixed OSCC cohorts and one study on TSCC in the Chinese population have reported a significant underexpression (16–18). This variation in the expression may be due to the influence of tobacco or the low basal expression level in the Indian population (17). However, limited studies are available on the association between H19 and their role in the prognosis of OSCC. Furthermore, there are no studies on the correlation between the expression of H19 and loco-regional recurrence/relapse of OSCC. Hence, the present study was carried out to analyze the expression of H19 correlated with clinicopathological parameters, tobacco habit, loco-regional recurrence, and overall survival.

## 2 Materials and methods

A total of 96 formalin-fixed paraffin-embedded (FFPE) OSCC tissues, as well as 30 matched adjacent normal mucosa (NM) FFPE tissues, were retrieved from the Department of Oral Pathology and Microbiology, SDM College of Dental Sciences and Hospital, Dharwad, Karnataka, India. These tissues were collected from patients who underwent treatment by surgery between 2015 and 2018 and stored at the SDM Craniofacial Surgery Unit.

### 2.1 Inclusion and exclusion criteria

The current study consisted of OSCC patients who had undergone radical neck dissection (RND) or modified radical neck dissection (MRND), with or without adjunct radiation or radiochemotherapy, and had at least 3 years of follow-up. Exclusion criteria included neoadjuvant radio and chemotherapy, immunodeficiency or other cancers, distant metastases, and inadequate data/follow-up.

All the clinical details, treatment, and follow-up details were collected. Histopathological evaluation of all the samples was carried out to confirm tumor content (≥80%) and also record the histopathological features.

### 2.2 Ethical clearance

Institutional Ethical Committee approval was obtained to conduct the study (Ref. No IRB. No. 2020/S/OP/71). Written informed consent was obtained from OSCC patients, excluding those who were deceased patients who could not be traced or contacted.

### 2.3 RNA isolation and quantitative reverse-transcription PCR

The tissue samples were retrieved from the Department of Oral Pathology and Microbiology, and the FFPE blocks were stored in the dark at room temperature (24 ± 2°C). An experienced oral pathologist performed histological evaluations on all samples to confirm OSCC diagnosis and tumor content (≥80%). Sections of 6–8 μm thickness from FFPE tissue blocks of OSCC and NM were taken using a microtome, and total RNA isolation was carried out as per our previously published optimized modified TRI reagent protocol (19). The tissues collected were subjected to qRT-PCR to determine the H19 expression.

### 2.4 Primer design

Primer sequences for H19 and GAPDH (endogenous control gene) were custom-designed using a software tool.^1^ Primer specificity was confirmed using the BLAST tool.^2^ The primer sequences were as follows (H19 forward: 5′-AGACACCATCGGAACAGCA-3′, H19 reverse: 3′-CTCTGGGATGATGTGGTGGC-5′) and glyceraldehyde 3-phosphate dehydrogenase (GAPDH) as the endogenous control gene (GAPDH forward: 5′-GGGGAAGGTGAAGGTCGGAG-3′, GAPDH reverse: 3′-ACGGTGCCATGGAATTTGCC-5′).

### 2.5 cDNA synthesis and real-time quantitative PCR

The extracted RNA was prepared at a concentration of 1 μg/μL per sample and transcribed into cDNA using the PrimeScript first-strand cDNA synthesis kit (TaKaRa, Dalian, China) following the manufacturer’s instructions. cDNA samples were diluted (1:10) with nuclease-free water and stored at −20°C until further use.

Quantitative reverse-transcription PCR was performed using PrimeScript RT Master Mix (TaKaRa, Dalian, China) in a total volume of 20 μL. Each experiment was carried out in triplicate. The LightCycler melting curve analysis yielded single product-specific melting temperatures. The PCR conditions were as follows: initial denaturation −95°C for 5 min, denaturation at −95°C for 5 s, and annealing at −60°C for 10 s. Amplicons were analyzed by high-resolution melting (HRM) at 1°C increment from 60°C to 95°C to determine the absolute melt curve of each amplicon. No primer dimers were generated during the 40 real-time PCR amplification cycles. The specificity of RT-qPCR products was confirmed using high-resolution gel electrophoresis, which produced a single product of the desired length.

The relative lncRNA expression levels were calculated using the 2^−ΔΔCt^ method provided by Schmittgen et al. (20). H19 expression levels were categorized into low-expression and high-expression groups, which were then compared to clinicopathological factors, habits, loco-regional recurrence, and overall survival.

### 2.6 Bioinformatics analysis

The relative expression of H19 levels in the Cancer Genome Atlas (TCGA) dataset was derived using the UCSC Xena platform for head and neck squamous cell carcinomas (HNSCCs). The R computer language’s ggplot2 package (v 3.3.5) was used to generate the TCGA box plots. The dplyr package (v 1.0.9) was also used for data-wrangling activities, including filtering, sorting, and summarizing the data before analysis.

### 2.7 Statistical analysis

The experimental data were expressed as mean ± SD. The statistical significance of differences in the resulting data was analyzed using GraphPad Prism (version 10.2.0, Boston, United States) and SPSS software (version 21.0., Armonk, NY), with *α* = 0.05, and a *p* value of <0.05 was considered statistically significant. The difference in the expression of H19 in OSCC and NM, as well as the expression-associated clinicopathological features, were determined using the *t*-test and ANOVA. There was no suitable cutoff value available for the expression of H19 by drawing the ROC curve. Hence, the mean value was taken as the cutoff value to determine the low and high expressions. Kaplan–Meier survival analysis followed by the log-rank test was performed. Multivariate Cox proportional hazards regression analysis was conducted to assess the ability of H19 as a predictor of loco-regional recurrence and overall survival for OSCC.

## 3 Results

### 3.1 Demographic and clinicopathological data

A total of 96 OSCC patients who have undergone surgical resection were considered for the study. The mean age of the patients was approximately 50 years. The majority of the patients were men, with the most common site being the buccal mucosa, particularly in the gingivobuccal sulcus (GBS), which was associated with tobacco chewing habits. Two-thirds of the cases were in the higher TNM stages (III and IV). All cases were histopathologically proven cases of OSCC, with 62.5% being well-differentiated. Approximately 50% of the cases showed positive lymph node metastasis and were treated with surgery followed by radiotherapy. A total of 66.7% of the cases exhibited loco-regional recurrence with a mean recurrence period of 16.66 months (Supplementary material 1; Table 1).

**Table 1:** Demographic and clinicopathological details of oral squamous cell carcinoma cases.

Parameter		Frequency	Percentage (%)
Age (years)	Mean ± SD	50 ± 11.17	-
	<50	46	47.91
	≥50	50	52.08
Sex	Male	86	89.6
	Female	10	10.4
Anatomical site	BM with or without GBS	70	72.9
	Tongue	19	19.7
	Hard palate	3	3.1
	Alveolus and lip	4	4.1
Habit	No habit	12	12.5
	Tobacco chewing	42	43.8
	Smoking	16	16.7
	Chewing and smoking	22	22.9
	Chewing + smoking + alcohol	4	4.2
Duration of Habit	<15 years	52	54.1
	≥15 years	44	45.8
TNM	Stage I	11	11.5
	Stage II	11	11.5
	Stage III	53	55.2
	Stage IV	21	21.9
Histopathology grade	Well-differentiated	60	62.5
	Moderately differentiated	28	29.1
	Poorly differentiated	08	8.3
PNI	No	67	69.8
	Yes	29	30.2
PVI	No	84	87.5
	Yes	12	12.5
Lymph node metastasis	No	51	53.1
	Present	45	46.9
Treatment	Surgery	24	25
	Surgery + Radiotherapy	47	48.9
	Surgery + Chemotherapy + Radiotherapy	25	26
Recurrence status	No recurrence	64	66.7
	Yes	32	33.3

### 3.2 Underexpression of H19 in OSCC cases and TCGA database

**Figure 1:**
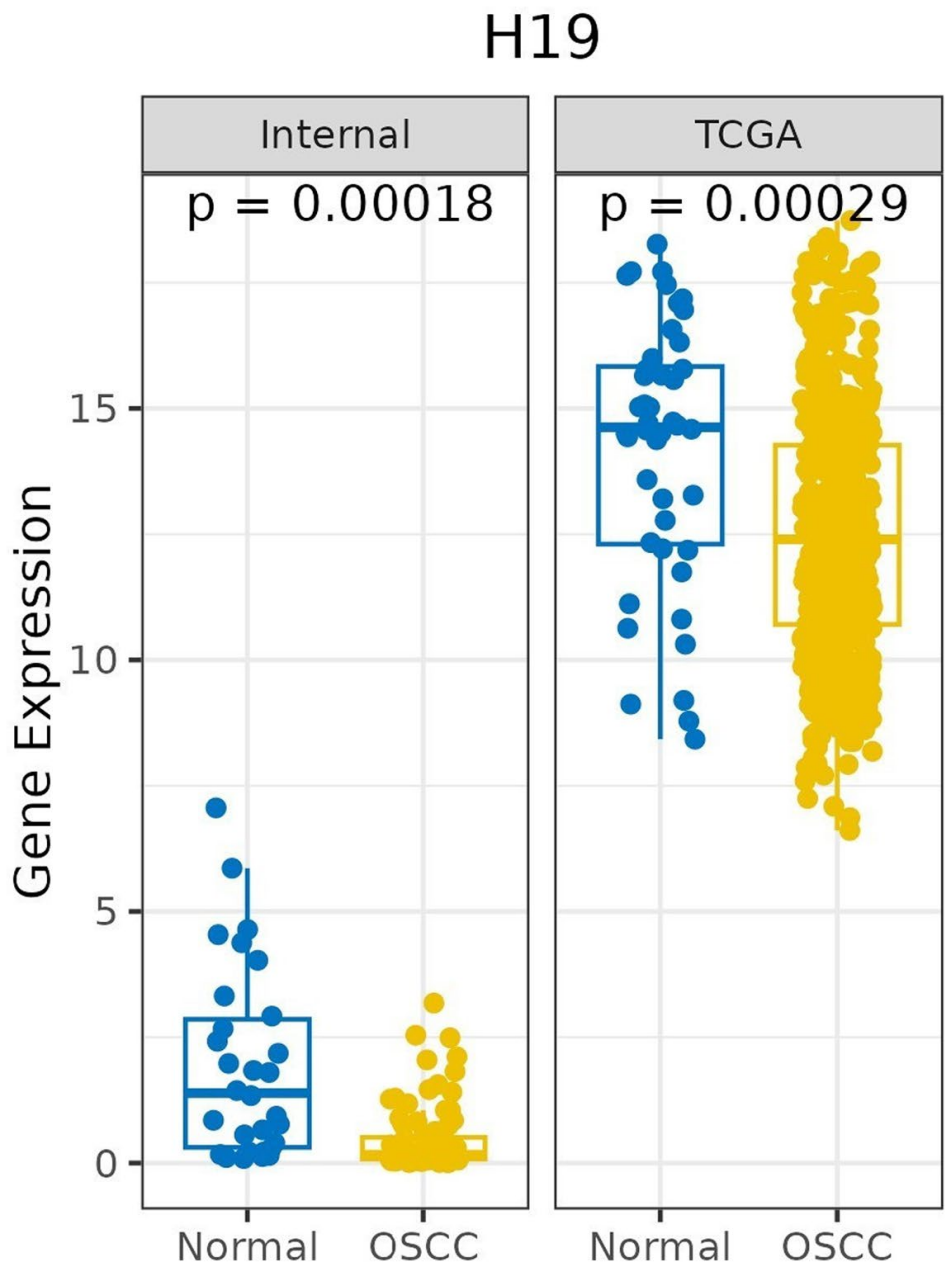
Box plot shows the comparison of relative expression of H19 in OSCC compared to normal mucosa in study cases (internal) (OSCC, *n* = 96 and normal *n* = 30) and TCGA database (OSCC, *n* = 517 and normal, *n* = 44).

The expression of H19 was measured in 96 OSCC tissues (average fold change 0.43) and compared to 30 NM tissues (average fold change 1.93). The H19 expression was significantly lower in the OSCC tissues than in the NM tissues (*p* < 0.001; unpaired test). Similar results were found when the H19 expression was downregulated in OSCC cases from the TCGA database (*n* = 517) compared to normal mucosa from the TCGA database (*n* = 44), with a *p* value <0.001 (unpaired’ test) (Figure 1; Supplementary material 2).

### 3.3 The relationship between H19 expression and tobacco habit and clinicopathological features in OSCC

The expression of H19 in the OSCC tissues was compared to clinicopathological characteristics and risk habits using the *t*-test and ANOVA. Only tobacco smoking was significantly correlated with H19 underexpression (*t*-test, *p* = 0.033), while none of the other clinicopathological characteristics were significantly correlated with the H19 expression (Table 2).

**Table 2:** Correlation of H19 expression with clinicopathological features and tobacco habit.

Parameter	*p* value (*—*t*-test, #—ANOVA)
Chewing tobacco	0.374*
Smoking tobacco	0.033*
Lymph node metastasis	0.236*
PNI	0.237*
PVI	0.088*
Site	0.108^#^
TNM grading	0.419^#^
H/P grading	0.665^#^

### 3.4 Underexpression of H19 is correlated with poor overall survival and positive loco-regional recurrences in OSCC patients

The receiver operating characteristic (ROC) curve and Youden index analysis (Youden index = sensitivity + specificity − 1) did not provide an acceptable cutoff value. Therefore, a mean H19 expression of 0.43 was used as the cutoff value for distinguishing high and low H19 expression.

**Figure 2:**
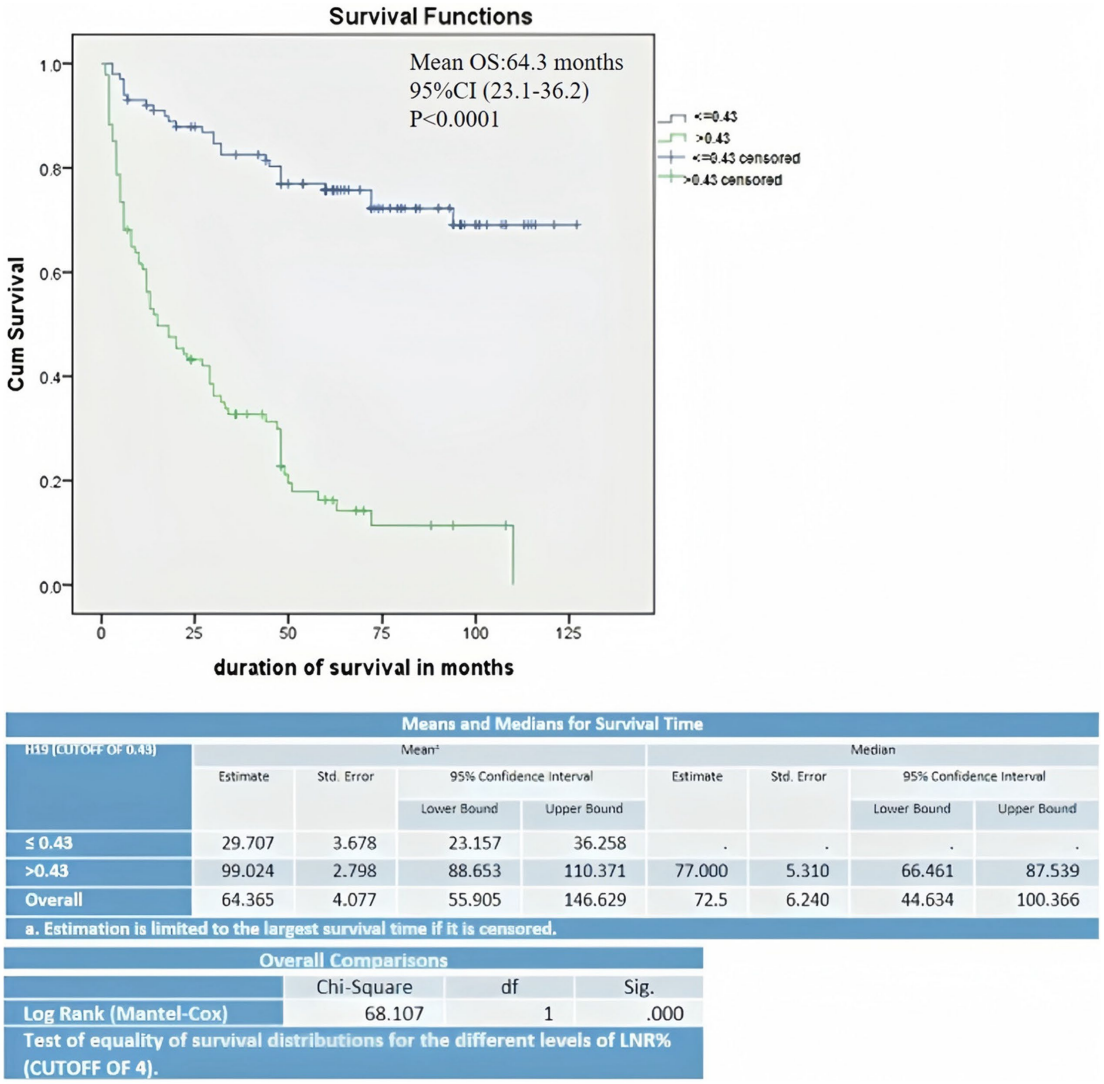
Kaplan–Meier curve and log-rank (Mantel–Cox) test survival estimate of OSCC cases correlated with H19 expression.

The Kaplan–Meier curve and log-rank (Mantel–Cox) test for overall survival showed that lower expression of H19 (≤ 0.43) was significantly associated with a lower mean OS period of 29.7 months compared to higher H19 expression (>0.43), which had a mean OS 99 months (*p* < 0.001) (Figure 2). Multivariate Cox’s proportional hazards regression analysis indicated that low H19 expression and positive lymph node metastasis were independent predictors of overall survival for OSCC (Table 3). Higher age, higher TNM staging, and low H19 expression were independent predictors of loco-regional recurrence (Table 4).

**Table 3:** Multivariable Cox regressions analysis of risk factors affecting the overall survival in oral squamous cell carcinomas.

		Multivariate survival analysis			
		HR	95.0% CI for HR		*p* value
			Lower	Upper	
Age	AGE (<50 years ≥50 years)	0.640	0.280	1.459	0.288
Sex	(Female vs. Male)	0.281	0.045	1.742	0.173
Habits	No habits	Reference category			
	Tobacco chewing	0.849	0.236	3.050	0.802
	Smoking	0.499	0.096	2.586	0.408
	Tobacco chewing and smoking	0.808	0.199	3.272	0.765
	Chewing + smoking + alcohol	0.000	0.000	-	0.974
Duration of Habit	Habit (<15 years ≥15 years)	2.068	0.878	4.867	0.096
Site	BM with or without GBS	Reference category			
	Tongue	0.139	0.036	0.535	0.064
	Hard palate	0.564	0.047	6.774	0.652
	Alveolus and lip	0.906	0.100	8.173	0.930
TNM staging (AJCC, 7th edition)	STAGE I	Reference category			
	STAGE II	0.646	0.134	3.103	0.585
	STAGE III	0.639	0.168	2.428	0.511
	STAGE IVa	1.220	0.288	5.177	0.787
Histopathology grading	Well-differentiated	Reference category			
	Moderately differentiated	0.702	0.283	1.738	0.444
	Poorly differentiated	0.837	0.621	1.82	0.267
PNI	PNI (yes vs. No)	1.379	0.569	3.347	0.477
PVI	PVI (yes vs. No)	0.430	0.106	1.741	0.237
Lymph node metastasis	(Yes vs. No)	4.396	1.775	10.886	0.001
Treatment	Surgery	Reference category			
	Surgery + Radiotherapy	0.848	0.337	2.134	0.726
	Surgery + Radiotherapy + Chemotherapy	0.923	0.313	2.726	0.885
Locoregional recurrence	Locoregional recurrence status (Yes vs. No)	0.721	0.331	3.125	0.932
H19	<0.43 vs. ≥ 0.43	0.373	0.147	0.944	0.037

**Table 4:** Multivariable Cox regressions analysis of risk factors affecting the loco-regional recurrence in oral squamous cell carcinomas.

	HR	95.0% CI for Exp(B)		Sig.
		Lower bound	Upper bound	
Age recorded (<50 years ≥50 years)	0.954	0.918	0.991	0.016
Sex (Female vs. Male)	3.982	0.426	0.226	0.226
Habit (Habit 1 vs. Habit 2 vs. Habit 3 vs. Habit 4)	4.504	0.473	0.191	0.191
Duration of habit (<15 years ≥15 years)	4.58	0.327	0.376	0.376
Site (Site 1 vs. Site 2 vs. Site 3 vs. Site 4)	3.558	0.742	0.112	0.112
TNM staging (Stage I vs. Stage II vs. Stage III vs. Stage III vs. Stage IV)	1.707	1.039	2.806	0.002
Histopathology (Well vs. Moderate vs. Poor)	0.357	0.038	0.368	0.368
PNI (Yes vs. No)	1.058	0.145	0.956	0.956
PVI (Yes vs. No)	3.762	0.569	0.246	0.246
Lymph node metastasis (Yes vs. No)	0.261	0.031	0.215	0.215
Treatment (Treatment1 vs. Treatment 2 vs. Treatment3 vs. treatment4)	4.877	0.403	0.213	0.213
H19 (<0.43 vs. ≥ 0.43)	0.110	0.027	0.452	0.002

*p < 0.05 is statistically significant. Normal: Normal mucosa; OSCC, Oral squamous cell carcinoma; TCGA: The Cancer Genome Atlas.

## 4 Discussion

LncRNA H19 is paternally imprinted and maternally expressed (21), located on chromosome 11p15.5, which is frequently linked to many diseases and a higher incidence of tumorigenesis. H19 has a length of 2.3 kb and consists of five exons and four introns (22–24).

H19 is known to have dual roles, functioning both as an oncogenic and tumor suppressor. H19 has been shown to have tumorigenic properties in breast cancer (10), hepatocellular carcinoma (HCC) (12), and ovarian carcinoma (25). However, other studies have demonstrated the tumor suppressor function of H19 in Wilms tumor and rhabdomyosarcoma (26). Studies have shown both oncogenic and tumor suppressor function of H19 in papillary thyroid carcinoma (27).

Only a few studies have addressed the role of H19 in OSCCs, and the results are inconsistent, similar to findings in other cancers. Additionally, the role of risk habit in the expression of H19 has not been elaborately addressed. Hence, the present study analyzed the expression of lncRNA H19 and its prognostic significance in OSCCs.

In the present study, H19 was significantly downregulated in the OSCC tissues compared to adjacent normal mucosa (NM) (Figure 1). The underexpression of H19 was significantly related to the tobacco smoking habit (Table 2). Some researchers, including Hong et al. (8), Zhang et al. (14), and Kou et al. (15), have shown overexpression of H19 in tumor tissues and cell lines compared to normal tissues. Conversely, the two lncRNA studies on OSCC among the Indian population have shown conflicting results on H19 expression related to risk habits (16, 17). Vishwakarma et al. (17) demonstrated significant downregulation of H19 in OSCC cases compared to adjacent normal mucosa, negatively correlated with tobacco smoking habit, consistent with our findings. In contrast, Arunkumar et al. (16) showed overexpression of H19 in OSCC but significantly downregulated in patients with a tobacco chewing habit. Piao et al. (18) studied a small cohort (*n* = 22) of tongue squamous cell carcinoma (TSCC) cases in a Chinese population and found underexpression of H19, consistent with our findings. This study also showed that overexpression of H19 was associated with reduced proliferation and migration in cell lines, but H19 expression was not correlated with risk habits.

The significant aspects of these findings are that studies showing overexpression of H19 were primarily focused on TSCC or included a majority of TSCC cases (Hong et al., 54.7% cases of TSCC) (8) and did not correlate risk habits with the expression of H19. In the two Indian studies (16, 17) including ours, the majority of OSCC cases were from the buccal mucosa, examining H19 expression concerning tobacco use, which revealed underexpression. Thus, H19 expression may be site-specific or related to tobacco use. However, Piao et al. (18) found that the downregulation of H19 in TSCC was not linked with risk habit, but their study used a small cohort.

The TCGA data analysis in the present study showed significant underexpression of H19 in OSCC cases compared to normal tissues (Figure 1), as also reported by Vishwakarma et al. (17) across pan-cancer types, suggesting a tumor suppressor function for H19. H19 expression was not correlated with any clinicopathological factors in the present study. Previous studies have observed that H19 overexpression was associated with late-stage TSCC (8, 14, 15) and positively associated with lymph node metastasis status (14, 15).

In the present study, the downregulation of H19 was significantly related to overall survival and loco-regional recurrence. Multivariate analysis showed that lymph node metastasis and low H19 expression were independent predictors of poor survival. Similarly, higher age group, TNM staging, and lower H19 expression were independent predictors of loco-regional recurrence. Although a few studies have demonstrated that increased H19 expression is associated with lower survival (8, 14), these studies did not perform a multivariate analysis adjusting for confounding factors. No investigations have been conducted on H19 expression and loco-regional recurrence in OSCC to compare with our findings.

The specific mechanism by which H19 downregulation leads to the development and invasion of OSCC is not known. H19 is highly expressed in the embryo, downregulated after birth, and reappears in tumors (28). Previous studies have linked H19 to many fundamental pathways of carcinogenesis as reviewed by Ghafouri-Fard et al. (23) and Matouk et al. (29), such as the p53 and HIF-1α, hedgehog, TGF-*β*, BcrAbl, RB-E2F1, HGF (MET), and Wnt/β-catenin signaling pathways. Apart from this, H19 also functions as ceRNAs to silence target mRNAs by sponging target miRNAs such as inhibiting miR-138 and facilitating EZH2 expression in OSCC8 or by sponging miRNA let-7a, the key regulator of tumor metastasis HMGA2, playing a critical role in the regulation of TSCC migration and invasions (15). Zhang et al. (30) found that H19 was underexpressed in HCC tumor tissues compared to peritumoral tissues. H19 inhibited tumor metastasis in HCC by modulating the miR-200 pathway and inducing mesenchymal-to-epithelial transition (MET).

Cigarette smoke (nicotine) has been demonstrated in lung-on-a-chip models to cause EMT by breaking down intercellular connections and initiating the process (31) and lncRNAs have been linked to the nicotine pathway (32). H19 downregulation discovered in smokers in this study could be responsible for the maintenance/activation of EMT in OSCCs. The oncogenic effects of cigarette smoke extend beyond EMT to the PI3K/AKT/mTOR signaling pathway regulated by H19 through a competing endogenous RNA network. The PI3K/AKT/mTOR signaling pathway influences Janus kinase signal transducer and activator of transcription (JAK–STAT) and NF-κB signaling pathways, affecting autophagy, apoptosis, translation, cell cycle regulation, nutrient uptake, fatty acid synthesis, and nuclear protein organization. It is also known to control angiogenesis in tumor cells. The PI3K protein, together with the AKT and mTOR proteins, are key players in this interaction. PI3K is activated by phosphorylation, produced by cell surface receptor signals and ligands such as cytokine receptors, GPCRs, RTKs, and integrins (33) Hence, tobacco, specifically smoking, may influence the expression of H19.

## 5 Conclusion

The current study’s findings show that H19 is a novel biomarker for overall survival and relapse, which could provide a therapeutic strategy for the targeted treatment of OSCC. Tobacco smoking, the most common cause of OSCCs, may contribute to H19 downregulation, resulting in OSCC development and invasion, although the exact process is unknown.

## 6 Limitations and recommendations for further studies

The current study does not examine H19 expression levels at the *in vitro* cell level or establish the role of H19 in the formation and progression of OSCC via regulatory signaling pathways. Furthermore, given the differences in H19 expression findings and the scarcity of data, more studies on clinical samples and cell lines are required to determine the role of H19 in OSCCs.

## Data Availability

The original contributions presented in the study are included in the article/Supplementary material, further inquiries can be directed to the corresponding authors.

## Footnotes

^1^https://bioinfo.ut.ee/primer3-0.4.0/

^2^https://blast.ncbi.nlm.nih.gov/Blast.cgi
