# Supplementary material for: Long non-coding RNA H19 as a prognostic biomarker for oral squamous cell carcinoma
Source: Front Med (Lausanne). 2024 Nov 26;11:1456963. doi: 10.3389/fmed.2024.1456963 (PMC11628285; doi:10.3389/fmed.2024.1456963)
Supplement: Supplementary file 1 [file Table_1.docx]

Table S1: Descriptive Analysis:

|  | N | Minimum | Maximum | Mean | Std. Deviation |
| --- | --- | --- | --- | --- | --- |
| AGE | 96 | 20.00 | 70.00 | 50.00 | 11.17 |
| Duration of habit | 96 | 0.00 | 48.00 | 14.89 | 8.73 |
| recurrence_months | 32 | 2.00 | 96.00 | 16.66 | 21.63 |
| Duration_of_survival_Months | 96 | 3.00 | 147.00 | 45.48 | 29.65 |
| H19 | 96 | .01 | 3.18 | 0.43 | 0.63 |
| H19 (≤ 0.43) | 70 | 72.9% |  |  |  |
|  | 26 | 27.1% |  |  |  |
| H19 (>0.43) |  |  |  |  |  |
|  |  |  |  |  |  |
|  |  |  |  |  |  |
