# Supplementary material for: Long non-coding RNA H19 as a prognostic biomarker for oral squamous cell carcinoma
Source: Front Med (Lausanne). 2024 Nov 26;11:1456963. doi: 10.3389/fmed.2024.1456963 (PMC11628285; doi:10.3389/fmed.2024.1456963)
Supplement: Supplementary file 2 [file Table_2.docx]

**Supplement Data**

**TCGA Data comparisons**

Number of sample retrieved from TCGA were 517 OSCC and 44 normal (healthy patient data), corresponding to selected genes. Each sample’s values given table S2 along with sample ID.

Table S2. TCGA Raw data. Values in Transcript per million unit.

| **Sample** | **Types_samples** | **H19** | **MEG3** | **MALAT1** | **HOTAIR** |
| --- | --- | --- | --- | --- | --- |
| TCGA-CR-7383-01 | OSCC | 12.89 | 4.7937 | 8.7292 | 2.3145 |
| TCGA-CR-7398-01 | OSCC | 14.859 | 5.4637 | 9.7549 | 7.6401 |
| TCGA-CV-A6JT-01 | OSCC | 14.0154 | 7.6171 | 11.123 | 0 |
| TCGA-HD-8635-01 | OSCC | 15.6685 | 8.447 | 11.0238 | 4.369 |
| TCGA-CR-6481-01 | OSCC | 11.5983 | 2.2662 | 9.1745 | 1.0281 |
| TCGA-CR-7395-01 | OSCC | 14.738 | 4.0776 | 10.1455 | 0 |
| TCGA-BB-7863-01 | OSCC | 9.9474 | 3.2478 | 10.6518 | 2.0833 |
| TCGA-CV-7427-01 | OSCC | 8.8944 | 0.6036 | 10.2534 | 2.7474 |
| TCGA-CV-6436-01 | OSCC | 9.9599 | 2.9505 | 11.4752 | 3.0516 |
| TCGA-CN-A63V-01 | OSCC | 13.8204 | 8.4012 | 9.9722 | 4.2006 |
| TCGA-BA-A6DE-01 | OSCC | 16.5618 | 2.7441 | 10.7116 | 2.8188 |
| TCGA-CV-7177-01 | OSCC | 10.9104 | 3.7783 | 8.6185 | 0 |
| TCGA-P3-A6T4-01 | OSCC | 6.6104 | 6.0174 | 12.6519 | 4.2703 |
| TCGA-DQ-5631-01 | OSCC | 10.3569 | 2.2629 | 9.7347 | 5.7345 |
| TCGA-CQ-A4CA-01 | OSCC | 15.7201 | 7.9152 | 10.636 | 0 |
| TCGA-CR-7365-01 | OSCC | 13.0824 | 3.5781 | 9.6051 | 2.5621 |
| TCGA-F7-A61W-01 | OSCC | 14.5635 | 7.5008 | 9.3805 | 2.7352 |
| TCGA-CV-6937-01 | OSCC | 11.7377 | 7.4816 | 10.9503 | 4.5168 |
| TCGA-CQ-7069-01 | OSCC | 9.8369 | 7.7693 | 11.2368 | 3.8078 |
| TCGA-D6-A74Q-01 | OSCC | 15.0962 | 5.7621 | 10.2121 | 4.4743 |
| TCGA-KU-A66T-01 | OSCC | 12.0698 | 7.1089 | 11.3841 | 4.2156 |
| TCGA-CN-4728-01 | OSCC | 12.8871 | 7.3822 | 11.395 | 1.502 |
| TCGA-CR-7401-01 | OSCC | 13.4274 | 4.6748 | 9.5453 | 3.298 |
| TCGA-CN-A63T-01 | OSCC | 15.3621 | 5.734 | 10.481 | 0 |
| TCGA-CV-5976-01 | OSCC | 14.2136 | 4.1556 | 10.5373 | 2.9113 |
| TCGA-CV-7254-01 | OSCC | 11.4058 | 4.407 | 9.3929 | 2.472 |
| TCGA-BB-A6UO-01 | OSCC | 10.8 | 6.3342 | 11.4811 | 1.2473 |
| TCGA-CR-6470-01 | OSCC | 10.0515 | 3.2474 | 9.2669 | 0 |
| TCGA-CV-7437-01 | OSCC | 8.8249 | 3.6928 | 9.488 | 4.9768 |
| TCGA-CV-7411-01 | OSCC | 12.006 | 2.9832 | 10.6056 | 1.8705 |
| TCGA-D6-A6EP-01 | OSCC | 9.6595 | 11.5193 | 10.7785 | 4.3432 |
| TCGA-BB-A5HZ-01 | OSCC | 17.4227 | 5.6124 | 10.6749 | 0 |
| TCGA-CR-6480-01 | OSCC | 8.9649 | 1.5748 | 9.3222 | 1.9885 |
| TCGA-IQ-A61E-01 | OSCC | 10.4566 | 5.0001 | 10.12 | 3.7126 |
| TCGA-CV-7415-01 | OSCC | 11.5618 | 4.4732 | 10.1326 | 3.9685 |
| TCGA-CN-6988-01 | OSCC | 16.4688 | 3.9341 | 10.0969 | 0 |
| TCGA-CV-5444-01 | OSCC | 13.1304 | 6.9773 | 10.3963 | 3.5236 |
| TCGA-CQ-A4C7-01 | OSCC | 14.2095 | 6.4238 | 11.7201 | 3.3375 |
| TCGA-CN-5365-01 | OSCC | 14.5383 | 3.1297 | 10.3112 | 2.6943 |
| TCGA-DQ-5624-01 | OSCC | 13.1152 | 1.1927 | 9.4882 | 4.0936 |
| TCGA-CR-7370-01 | OSCC | 14.671 | 4.6195 | 10.8092 | 3.283 |
| TCGA-BA-5559-01 | OSCC | 14.3547 | 4.9395 | 10.5194 | 1.9363 |
| TCGA-CV-7247-01 | OSCC | 14.4417 | 2.8316 | 8.6071 | 3.8676 |
| TCGA-CR-7391-01 | OSCC | 12.0395 | 5.0181 | 9.5674 | 0.5687 |
| TCGA-CV-A6JU-01 | OSCC | 14.5802 | 8.7424 | 10.9874 | 0.9855 |
| TCGA-DQ-7591-01 | OSCC | 14.5742 | 2.826 | 11.0415 | 4.1904 |
| TCGA-T3-A92M-01 | OSCC | 10.3024 | 6.6944 | 12.259 | 4.2656 |
| TCGA-CN-5374-01 | OSCC | 9.948 | 4.8311 | 11.212 | 0 |
| TCGA-CR-7369-01 | OSCC | 13.8484 | 3.151 | 11.2681 | 1.7652 |
| TCGA-RS-A6TO-01 | OSCC | 11.0253 | 5.8125 | 11.6967 | 5.4128 |
| TCGA-BA-6868-01 | OSCC | 7.5945 | 3.5968 | 10.9363 | 4.4194 |
| TCGA-F7-7848-01 | OSCC | 13.9315 | 4.672 | 10.0776 | 0 |
| TCGA-CR-7376-01 | OSCC | 12.327 | 4.8478 | 9.1477 | 3.3443 |
| TCGA-CV-7421-01 | OSCC | 12.0709 | 5.3223 | 9.0483 | 3.0227 |
| TCGA-CQ-5325-01 | OSCC | 14.3497 | 4.1415 | 9.4905 | 0.8737 |
| TCGA-CV-7433-01 | OSCC | 12.4596 | 4.3182 | 8.9688 | 3.3066 |
| TCGA-CN-6012-01 | OSCC | 11.3976 | 4.5641 | 10.1618 | 1.9381 |
| TCGA-CV-A45Z-01 | OSCC | 14.5298 | 6.0078 | 10.7934 | 3.8619 |
| TCGA-BA-A6DG-01 | OSCC | 13.9991 | 7.8415 | 11.7364 | 2.7746 |
| TCGA-CN-4729-01 | OSCC | 10.2988 | 5.3969 | 11.5096 | 2.4778 |
| TCGA-CV-5979-01 | OSCC | 13.3623 | 5.5203 | 10.0694 | 0 |
| TCGA-CV-A45U-01 | OSCC | 11.6004 | 7.4343 | 12.325 | 4.3505 |
| TCGA-DQ-7590-01 | OSCC | 10.7419 | 5.4315 | 10.4417 | 1.6166 |
| TCGA-CR-7388-01 | OSCC | 10.7424 | 4.3949 | 11.1779 | 2.9036 |
| TCGA-BA-A4IG-01 | OSCC | 12.1473 | 1.4234 | 10.8322 | 0 |
| TCGA-P3-A5Q6-01 | OSCC | 11.0565 | 6.7163 | 10.2888 | 3.3755 |
| TCGA-CV-7180-01 | OSCC | 13.3096 | 2.3206 | 10.3595 | 1.9463 |
| TCGA-CR-6492-01 | OSCC | 9.3701 | 5.3134 | 10.8966 | 2.4805 |
| TCGA-CN-6992-01 | OSCC | 10.5292 | 5.2088 | 9.4331 | 0.3676 |
| TCGA-CR-7373-01 | OSCC | 14.1181 | 2.9476 | 9.908 | 1.9943 |
| TCGA-CR-7371-01 | OSCC | 12.0426 | 3.306 | 9.6093 | 2.0706 |
| TCGA-BA-4074-01 | OSCC | 14.0652 | 6.8464 | 9.6149 | 2.0297 |
| TCGA-CV-7435-01 | OSCC | 12.52 | 5.5512 | 9.0735 | 0.4672 |
| TCGA-DQ-7595-01 | OSCC | 17.3083 | 1.4481 | 8.6515 | 0 |
| TCGA-BA-A6DB-01 | OSCC | 14.4081 | 7.7131 | 10.8742 | 2.9859 |
| TCGA-CN-6021-01 | OSCC | 10.9426 | 1.7454 | 9.6355 | 2.5125 |
| TCGA-D6-6825-01 | OSCC | 12.2472 | 3.9996 | 11.608 | 1.7518 |
| TCGA-CN-6998-01 | OSCC | 12.665 | 1.9412 | 9.744 | 3.0799 |
| TCGA-CV-6948-01 | OSCC | 12.2344 | 5.2481 | 12.3512 | 4.2972 |
| TCGA-UF-A7JC-01 | OSCC | 11.2148 | 7.6177 | 11.0931 | 2.5918 |
| TCGA-MT-A7BN-01 | OSCC | 17.7962 | 8.478 | 10.8787 | 6.4721 |
| TCGA-BA-A6DA-01 | OSCC | 8.3805 | 6.2464 | 11.9012 | 1.6652 |
| TCGA-CV-A468-01 | OSCC | 13.1471 | 7.177 | 12.2684 | 1.6679 |
| TCGA-UF-A71E-01 | OSCC | 9.6622 | 4.7535 | 11.6638 | 3.6083 |
| TCGA-HD-7754-01 | OSCC | 13.3378 | 4.6899 | 9.7482 | 0 |
| TCGA-D6-A6ES-01 | OSCC | 8.8395 | 4.45 | 9.2288 | 0 |
| TCGA-CV-7434-01 | OSCC | 13.1901 | 2.5867 | 10.0656 | 3.1841 |
| TCGA-IQ-A61I-01 | OSCC | 10.3536 | 2.8318 | 11.6828 | 1.4586 |
| TCGA-CN-4736-01 | OSCC | 14.8861 | 6.3744 | 11.2805 | 3.2075 |
| TCGA-BA-7269-01 | OSCC | 11.6461 | 6.0634 | 9.4104 | 1.3203 |
| TCGA-CV-6433-01 | OSCC | 9.4117 | 3.0676 | 10.9516 | 2.0981 |
| TCGA-CR-7385-01 | OSCC | 7.9239 | 3.4012 | 10.7522 | 0 |
| TCGA-CV-6441-01 | OSCC | 18.3981 | 1.8511 | 9.8374 | 4.534 |
| TCGA-CV-5978-01 | OSCC | 9.0764 | 5.0813 | 12.0136 | 3.1319 |
| TCGA-CN-6017-01 | OSCC | 16.9145 | 6.9528 | 10.2495 | 1.7258 |
| TCGA-CN-5367-01 | OSCC | 14.0928 | 7.2474 | 9.9249 | 1.7296 |
| TCGA-CV-7252-01 | OSCC | 12.382 | 3.9007 | 9.6802 | 3.4914 |
| TCGA-QK-A6IG-01 | OSCC | 15.9984 | 6.1078 | 10.7588 | 2.1846 |
| TCGA-CV-A45R-01 | OSCC | 12.7893 | 5.8526 | 11.4025 | 4.1957 |
| TCGA-UF-A7JV-01 | OSCC | 8.6498 | 5.6611 | 11.0883 | 1.8842 |
| TCGA-CX-7219-01 | OSCC | 10.9778 | 4.6118 | 9.632 | 2.6182 |
| TCGA-BA-A6D8-01 | OSCC | 8.806 | 8.2303 | 10.0493 | 0 |
| TCGA-CR-7382-01 | OSCC | 15.1782 | 5.5437 | 9.7208 | 3.6963 |
| TCGA-CV-6953-01 | OSCC | 12.278 | 2.8969 | 11.3592 | 2.7663 |
| TCGA-CN-6022-01 | OSCC | 14.2106 | 7.4609 | 9.4622 | 4.143 |
| TCGA-CQ-6221-01 | OSCC | 12.6164 | 2.7897 | 8.7899 | 2.4426 |
| TCGA-HD-7753-01 | OSCC | 10.9038 | 3.4384 | 9.6115 | 3.3925 |
| TCGA-H7-8502-01 | OSCC | 10.283 | 7.349 | 11.2623 | 3.837 |
| TCGA-CN-6020-01 | OSCC | 10.1739 | 5.2731 | 10.1637 | 3.5251 |
| TCGA-P3-A5QA-01 | OSCC | 13.3318 | 4.9876 | 10.9406 | 3.242 |
| TCGA-CQ-7072-01 | OSCC | 16.2048 | 8.5757 | 11.6905 | 5.6724 |
| TCGA-CN-6016-01 | OSCC | 15.7972 | 5.7042 | 9.0624 | 4.0885 |
| TCGA-CR-5248-01 | OSCC | 7.8614 | 2.1439 | 10.4538 | 2.4321 |
| TCGA-D6-6823-01 | OSCC | 17.0594 | 3.4822 | 9.5146 | 3.2095 |
| TCGA-MT-A67F-01 | OSCC | 13.6078 | 5.7914 | 11.9925 | 0.6368 |
| TCGA-CN-4725-01 | OSCC | 14.263 | 5.6302 | 11.1914 | 1.7069 |
| TCGA-CV-A463-01 | OSCC | 9.303 | 2.5883 | 12.5297 | 4.053 |
| TCGA-BA-4078-01 | OSCC | 13.4231 | 8.0893 | 12.3935 | 4.1338 |
| TCGA-CV-7245-01 | OSCC | 14.8713 | 2.1864 | 8.6712 | 3.8097 |
| TCGA-QK-A8ZB-01 | OSCC | 14.5392 | 6.7169 | 12.096 | 3.7352 |
| TCGA-CN-5363-01 | OSCC | 12.5171 | 4.5019 | 8.87 | 4.4393 |
| TCGA-CV-A45P-01 | OSCC | 11.7294 | 5.0436 | 12.2604 | 2.9078 |
| TCGA-CV-7568-01 | OSCC | 12.4953 | 4.8529 | 13.2109 | 3.8093 |
| TCGA-CN-4722-01 | OSCC | 12.3474 | 5.4085 | 11.0808 | 3.2601 |
| TCGA-BB-4224-01 | OSCC | 10.6863 | 4.986 | 13.294 | 4.4938 |
| TCGA-CQ-5326-01 | OSCC | 9.883 | 6.6422 | 9.8517 | 2.9646 |
| TCGA-BB-7861-01 | OSCC | 8.4079 | 1.5435 | 10.8378 | 1.6935 |
| TCGA-CR-6487-01 | OSCC | 10.9929 | 4.1036 | 10.877 | 0.7689 |
| TCGA-BA-A6DI-01 | OSCC | 16.0596 | 6.831 | 11.8143 | 1.5586 |
| TCGA-CN-6994-01 | OSCC | 9.7049 | 5.8967 | 10.753 | 5.6209 |
| TCGA-P3-A6T6-01 | OSCC | 10.0553 | 5.3244 | 10.5418 | 0 |
| TCGA-CN-4739-01 | OSCC | 17.0937 | 5.7129 | 11.2735 | 0 |
| TCGA-DQ-7594-01 | OSCC | 11.6398 | 5.4052 | 10.0401 | 1.9924 |
| TCGA-CV-A45W-01 | OSCC | 12.609 | 5.8077 | 12.4712 | 2.7144 |
| TCGA-BA-5556-01 | OSCC | 12.1284 | 1.4259 | 8.4818 | 1.5966 |
| TCGA-QK-AA3K-01 | OSCC | 13.6654 | 10.3593 | 12.3908 | 5.6144 |
| TCGA-CN-4727-01 | OSCC | 17.7934 | 4.5276 | 10.9333 | 0.9703 |
| TCGA-BB-7864-01 | OSCC | 14.1034 | 5.1507 | 10.1535 | 0 |
| TCGA-CV-5966-01 | OSCC | 11.1913 | 4.7571 | 9.4033 | 2.3483 |
| TCGA-CR-6482-01 | OSCC | 11.5838 | 2.3459 | 9.7263 | 4.046 |
| TCGA-CV-6936-01 | OSCC | 7.7088 | 3.5104 | 10.2917 | 3.7909 |
| TCGA-CQ-5332-01 | OSCC | 15.1512 | 4.7327 | 9.273 | 2.8362 |
| TCGA-CN-4734-01 | OSCC | 9.8687 | 4.4171 | 10.138 | 1.5116 |
| TCGA-CQ-5327-01 | OSCC | 15.1003 | 2.7586 | 9.2667 | 2.5375 |
| TCGA-CV-A460-01 | OSCC | 11.1751 | 6.8151 | 10.6683 | 2.2974 |
| TCGA-UF-A71A-01 | OSCC | 13.9337 | 6.7844 | 11.1834 | 1.225 |
| TCGA-BA-A4IF-01 | OSCC | 15.4369 | 6.2448 | 10.4935 | 2.3811 |
| TCGA-F7-A50J-01 | OSCC | 12.9843 | 4.7921 | 13.0483 | 3.2749 |
| TCGA-CV-7100-01 | OSCC | 10.3696 | 4.1716 | 9.98 | 2.6325 |
| TCGA-CQ-A4C9-01 | OSCC | 13.6793 | 5.4815 | 10.1627 | 3.8948 |
| TCGA-CR-7374-01 | OSCC | 11.1111 | 5.9497 | 10.3968 | 3.7867 |
| TCGA-CV-A6JZ-01 | OSCC | 10.919 | 5.8773 | 11.5981 | 3.4024 |
| TCGA-D6-6827-01 | OSCC | 11.9261 | 4.1372 | 9.7214 | 8.1938 |
| TCGA-CQ-7068-01 | OSCC | 11.7222 | 2.3357 | 10.4778 | 4.9708 |
| TCGA-BA-5152-01 | OSCC | 9.6865 | 1.3294 | 9.0414 | 4.7067 |
| TCGA-CN-A49B-01 | OSCC | 10.4033 | 5.0667 | 11.0595 | 3.9685 |
| TCGA-CV-A6JN-01 | OSCC | 9.5603 | 5.7279 | 10.9579 | 2.663 |
| TCGA-CN-4723-01 | OSCC | 10.9926 | 7.617 | 12.0315 | 4.0999 |
| TCGA-CN-A6V7-01 | OSCC | 12.1833 | 1.464 | 11.742 | 0 |
| TCGA-HD-8634-01 | OSCC | 11.2286 | 4.6604 | 10.7717 | 0.6712 |
| TCGA-CV-6945-01 | OSCC | 11.6505 | 5.9404 | 12.2316 | 4.5065 |
| TCGA-DQ-7588-01 | OSCC | 13.712 | 1.6406 | 8.517 | 2.2667 |
| TCGA-DQ-5630-01 | OSCC | 11.7194 | 6.8905 | 9.9238 | 2.648 |
| TCGA-CV-7429-01 | OSCC | 11.715 | 5.6059 | 8.6486 | 2.2473 |
| TCGA-CN-4737-01 | OSCC | 16.5611 | 7.2991 | 12.0469 | 4.0337 |
| TCGA-D6-A6EQ-01 | OSCC | 12.5349 | 6.9056 | 11.0157 | 3.4869 |
| TCGA-D6-A4ZB-01 | OSCC | 11.4458 | 7.0609 | 12.7497 | 3.5087 |
| TCGA-CQ-5323-01 | OSCC | 14.805 | 4.2469 | 9.9221 | 3.4845 |
| TCGA-DQ-5625-01 | OSCC | 14.6005 | 4.5804 | 9.4301 | 2.6178 |
| TCGA-CV-A6K2-01 | OSCC | 8.4822 | 5.5403 | 11.0475 | 4.4783 |
| TCGA-IQ-A61G-01 | OSCC | 14.5924 | 3.7336 | 11.3339 | 3.521 |
| TCGA-BA-6869-01 | OSCC | 15.4948 | 3.589 | 11.3583 | 0 |
| TCGA-F7-A61S-01 | OSCC | 10.5964 | 3.3513 | 10.4779 | 4.2789 |
| TCGA-BB-7871-01 | OSCC | 11.1483 | 5.3322 | 10.7929 | 3.8286 |
| TCGA-CV-A6JE-01 | OSCC | 12.1068 | 8.2628 | 12.5534 | 4.0388 |
| TCGA-DQ-7589-01 | OSCC | 11.9697 | 6.6097 | 10.0017 | 3.2485 |
| TCGA-CN-4740-01 | OSCC | 14.3533 | 5.9528 | 9.7095 | 0 |
| TCGA-CV-A45V-01 | OSCC | 12.754 | 6.1265 | 11.4525 | 3.2751 |
| TCGA-UP-A6WW-01 | OSCC | 10.3056 | 5.6481 | 11.0135 | 3.0565 |
| TCGA-CN-4742-01 | OSCC | 11.3697 | 4.3573 | 9.7913 | 1.7997 |
| TCGA-CV-6942-01 | OSCC | 14.3452 | 6.0737 | 11.6408 | 2.1557 |
| TCGA-CR-6477-01 | OSCC | 11.0804 | 3.5656 | 8.5907 | 1.3818 |
| TCGA-CX-7085-01 | OSCC | 13.4899 | 6.1057 | 10.2715 | 0.5476 |
| TCGA-CR-6473-01 | OSCC | 11.6488 | 3.2048 | 10.2544 | 1.2449 |
| TCGA-BB-7872-01 | OSCC | 10.5483 | 1.8989 | 10.7118 | 3.6664 |
| TCGA-D6-6517-01 | OSCC | 8.8646 | 2.7338 | 9.8399 | 4.7513 |
| TCGA-CN-A641-01 | OSCC | 15.4973 | 3.692 | 9.4288 | 4.2549 |
| TCGA-IQ-A6SH-01 | OSCC | 10.466 | 3.7859 | 10.4115 | 2.8316 |
| TCGA-CV-7091-01 | OSCC | 11.7491 | 3.4989 | 10.0859 | 2.4803 |
| TCGA-CV-5430-01 | OSCC | 14.1369 | 4.5921 | 11.1366 | 2.3279 |
| TCGA-F7-8489-01 | OSCC | 9.7357 | 4.0335 | 11.272 | 1.7508 |
| TCGA-UF-A7JF-01 | OSCC | 11.9217 | 5.7115 | 11.5929 | 4.5666 |
| TCGA-IQ-7630-01 | OSCC | 9.341 | 5.4357 | 11.9354 | 4.3441 |
| TCGA-BA-4077-01 | OSCC | 10.3177 | 2.4144 | 9.512 | 1.7682 |
| TCGA-BB-7870-01 | OSCC | 17.8909 | 3.5469 | 9.9451 | 4.571 |
| TCGA-CR-6478-01 | OSCC | 12.1038 | 2.221 | 9.3746 | 2.221 |
| TCGA-CN-A642-01 | OSCC | 12.7663 | 4.8037 | 11.0762 | 5.0049 |
| TCGA-CN-6018-01 | OSCC | 12.3833 | 4.5811 | 9.6675 | 4.2125 |
| TCGA-CN-6010-01 | OSCC | 8.5094 | 4.4533 | 10.0073 | 2.6603 |
| TCGA-CV-A45Q-01 | OSCC | 17.4937 | 6.6349 | 11.0106 | 0 |
| TCGA-CQ-6222-01 | OSCC | 16.2138 | 7.7412 | 10.2545 | 2.1386 |
| TCGA-CR-5243-01 | OSCC | 9.9099 | 4.3573 | 10.1215 | 1.7454 |
| TCGA-D6-8568-01 | OSCC | 13.1898 | 6.1785 | 10.6412 | 0 |
| TCGA-P3-A6T3-01 | OSCC | 11.5864 | 5.231 | 11.9834 | 1.9471 |
| TCGA-C9-A480-01 | OSCC | 13.3306 | 3.9503 | 12.2049 | 1.8044 |
| TCGA-CR-6488-01 | OSCC | 13.0527 | 7.6501 | 12.1159 | 3.5165 |
| TCGA-DQ-7592-01 | OSCC | 15.0705 | 4.9227 | 9.6457 | 1.4879 |
| TCGA-UF-A71B-01 | OSCC | 9.7501 | 5.6589 | 10.5776 | 1.9434 |
| TCGA-T2-A6X2-01 | OSCC | 12.6448 | 5.3468 | 12.2351 | 3.396 |
| TCGA-CV-6940-01 | OSCC | 12.2828 | 4.4258 | 10.6716 | 3.0197 |
| TCGA-CV-7413-01 | OSCC | 12.7823 | 6.3464 | 10.8197 | 4.9062 |
| TCGA-CR-7404-01 | OSCC | 10.7871 | 5.3754 | 10.9289 | 0.963 |
| TCGA-CV-7432-01 | OSCC | 12.6667 | 1.8849 | 8.8268 | 1.3443 |
| TCGA-CV-5440-01 | OSCC | 14.6866 | 5.1581 | 11.9875 | 3.2745 |
| TCGA-CQ-A4CE-01 | OSCC | 13.1799 | 4.7661 | 12.9582 | 2.9704 |
| TCGA-CV-7422-01 | OSCC | 10.7136 | 5.3238 | 10.3534 | 1.8494 |
| TCGA-CV-6950-01 | OSCC | 14.1533 | 4.9655 | 9.1291 | 1.687 |
| TCGA-CV-A45X-01 | OSCC | 15.6299 | 4.369 | 11.6974 | 4.4077 |
| TCGA-CV-7263-01 | OSCC | 13.3176 | 4.1228 | 9.2777 | 2.0772 |
| TCGA-CV-7410-01 | OSCC | 12.0287 | 6.1551 | 11.0366 | 3.8056 |
| TCGA-CV-A45T-01 | OSCC | 13.1075 | 5.96 | 11.3151 | 2.9746 |
| TCGA-BB-4217-01 | OSCC | 14.7073 | 3.631 | 10.8767 | 2.8226 |
| TCGA-CV-7406-01 | OSCC | 15.2351 | 4.9168 | 8.8761 | 2.6577 |
| TCGA-CV-7089-01 | OSCC | 10.6524 | 2.5481 | 10.4227 | 2.6444 |
| TCGA-CV-A461-01 | OSCC | 16.8175 | 5.0579 | 11.0885 | 1.6188 |
| TCGA-D6-A6EK-01 | OSCC | 9.7146 | 4.4762 | 11.113 | 2.2317 |
| TCGA-CN-5359-01 | OSCC | 10.7268 | 5.7345 | 10.4441 | 1.5533 |
| TCGA-CQ-5330-01 | OSCC | 14.2585 | 6.0119 | 10.2646 | 2.1386 |
| TCGA-CV-5977-01 | OSCC | 17.5349 | 4.8883 | 10.6301 | 2.2677 |
| TCGA-BA-A4II-01 | OSCC | 10.1294 | 4.795 | 10.3589 | 0.92 |
| TCGA-CR-7402-01 | OSCC | 11.497 | 4.0091 | 10.7639 | 0 |
| TCGA-CV-5443-01 | OSCC | 11.336 | 4.6243 | 10.5657 | 2.3621 |
| TCGA-UF-A7JS-01 | OSCC | 15.9567 | 7.1898 | 10.9594 | 0 |
| TCGA-IQ-7632-01 | OSCC | 12.9678 | 2.8123 | 11.062 | 2.0996 |
| TCGA-CN-4730-01 | OSCC | 14.1804 | 4.1503 | 9.9791 | 0 |
| TCGA-C9-A47Z-01 | OSCC | 14.3862 | 4.6631 | 11.838 | 1.7065 |
| TCGA-CV-7101-01 | OSCC | 9.8431 | 3.3458 | 10.0176 | 2.3692 |
| TCGA-CQ-6227-01 | OSCC | 14.5754 | 4.8778 | 9.2454 | 1.829 |
| TCGA-CV-7253-01 | OSCC | 16.5378 | 1.178 | 8.4823 | 0 |
| TCGA-CV-A6K1-01 | OSCC | 14.6584 | 10.2019 | 11.8924 | 3.1094 |
| TCGA-CR-7377-01 | OSCC | 13.2437 | 3.9072 | 8.5104 | 2.4727 |
| TCGA-HD-7831-01 | OSCC | 13.1628 | 6.3239 | 9.942 | 3.356 |
| TCGA-CN-4735-01 | OSCC | 8.961 | 5.5084 | 10.8456 | 5.5205 |
| TCGA-BA-6872-01 | OSCC | 12.4101 | 3.3524 | 10.7046 | 1.7895 |
| TCGA-CV-7416-01 | OSCC | 9.2952 | 5.6863 | 10.772 | 3.2436 |
| TCGA-BB-A5HU-01 | OSCC | 8.5431 | 6.7303 | 11.5758 | 3.9394 |
| TCGA-CQ-A4C6-01 | OSCC | 12.2046 | 5.459 | 11.3083 | 2.2567 |
| TCGA-BB-A5HY-01 | OSCC | 15.2116 | 5.5322 | 11.3134 | 5.2892 |
| TCGA-CV-7099-01 | OSCC | 12.3064 | 3.0455 | 10.1007 | 2.1423 |
| TCGA-KU-A6H7-01 | OSCC | 9.8482 | 4.9903 | 10.7663 | 1.3897 |
| TCGA-BB-4225-01 | OSCC | 10.7328 | 1.2835 | 9.2992 | 5.7472 |
| TCGA-CV-6955-01 | OSCC | 8.2662 | 1.0882 | 9.9415 | 1.7015 |
| TCGA-BA-5153-01 | OSCC | 10.2122 | 1.9352 | 10.4744 | 0.5565 |
| TCGA-CN-A6V3-01 | OSCC | 15.3568 | 3.3721 | 10.6672 | 3.2065 |
| TCGA-CV-A6K0-01 | OSCC | 14.0218 | 6.4413 | 11.3552 | 1.326 |
| TCGA-BA-A6DJ-01 | OSCC | 11.7438 | 4.445 | 11.1636 | 2.8199 |
| TCGA-CN-4738-01 | OSCC | 12.4076 | 6.8847 | 12.1447 | 1.5589 |
| TCGA-CV-6960-01 | OSCC | 11.8447 | 3.1741 | 9.3329 | 4.1497 |
| TCGA-MZ-A6I9-01 | OSCC | 13.6027 | 4.649 | 10.1204 | 0 |
| TCGA-CQ-6228-01 | OSCC | 14.899 | 3.7308 | 11.6336 | 0 |
| TCGA-F7-A622-01 | OSCC | 15.8422 | 5.6461 | 11.2399 | 3.5814 |
| TCGA-CV-7236-01 | OSCC | 11.3098 | 4.6222 | 9.3951 | 2.6062 |
| TCGA-RS-A6TP-01 | OSCC | 13.7058 | 2.1797 | 10.7087 | 4.244 |
| TCGA-HD-A4C1-01 | OSCC | 9.57 | 6.0886 | 10.2386 | 3.4117 |
| TCGA-CV-7255-01 | OSCC | 18.1055 | 2.1848 | 9.6658 | 0.348 |
| TCGA-F7-8298-01 | OSCC | 11.7719 | 6.5304 | 10.1389 | 4.05 |
| TCGA-4P-AA8J-01 | OSCC | 14.3943 | 9.4884 | 11.6613 | 4.8751 |
| TCGA-BA-5558-01 | OSCC | 13.3523 | 3.1218 | 11.0516 | 2.5524 |
| TCGA-P3-A5QF-01 | OSCC | 16.9613 | 3.7138 | 10.7373 | 0.5521 |
| TCGA-CV-5973-01 | OSCC | 17.9329 | 4.7827 | 13.349 | 4.1357 |
| TCGA-BA-6871-01 | OSCC | 10.813 | 7.1349 | 10.7791 | 4.7226 |
| TCGA-QK-A8Z7-01 | OSCC | 15.1766 | 6.3735 | 10.9505 | 5.8086 |
| TCGA-CQ-A4CB-01 | OSCC | 10.5166 | 6.8213 | 11.9108 | 2.8641 |
| TCGA-CX-7086-01 | OSCC | 17.6545 | 1.2964 | 10.3948 | 1.065 |
| TCGA-CR-7390-01 | OSCC | 12.3554 | 4.693 | 9.5719 | 1.7204 |
| TCGA-P3-A6T5-01 | OSCC | 11.9458 | 5.6478 | 12.3021 | 4.5101 |
| TCGA-CV-7183-01 | OSCC | 14.5209 | 2.1767 | 9.4563 | 2.1767 |
| TCGA-CQ-A4CI-01 | OSCC | 9.3168 | 5.745 | 11.203 | 1.9983 |
| TCGA-HD-8224-01 | OSCC | 15.0072 | 6.4949 | 10.4924 | 0 |
| TCGA-QK-A8Z8-01 | OSCC | 9.929 | 4.5295 | 10.8026 | 3.5408 |
| TCGA-CN-5358-01 | OSCC | 13.6416 | 5.9105 | 10.504 | 1.6451 |
| TCGA-CN-6989-01 | OSCC | 13.3254 | 6.8652 | 9.602 | 2.8313 |
| TCGA-CV-6941-01 | OSCC | 16.6244 | 6.2293 | 10.5086 | 4.6135 |
| TCGA-BA-4075-01 | OSCC | 9.6775 | 6.7066 | 9.0369 | 2.8937 |
| TCGA-CR-6484-01 | OSCC | 15.6364 | 5.0953 | 9.8911 | 2.6576 |
| TCGA-CN-6024-01 | OSCC | 13.7087 | 5.5419 | 10.4124 | 2.6346 |
| TCGA-UF-A7JT-01 | OSCC | 14.0973 | 8.986 | 11.3295 | 4.0889 |
| TCGA-MT-A67D-01 | OSCC | 15.1674 | 6.3232 | 11.2904 | 3.0183 |
| TCGA-CR-7392-01 | OSCC | 15.8216 | 4.9754 | 9.8423 | 3.065 |
| TCGA-CR-7379-01 | OSCC | 14.8611 | 3.5603 | 8.6279 | 2.2013 |
| TCGA-CX-7082-01 | OSCC | 11.5582 | 4.7744 | 10.8417 | 2.8604 |
| TCGA-H7-A76A-01 | OSCC | 10.0926 | 8.6419 | 12.3983 | 3.7279 |
| TCGA-P3-A6T7-01 | OSCC | 11.8983 | 7.9065 | 12.2705 | 2.1419 |
| TCGA-CN-4733-01 | OSCC | 12.4833 | 3.7907 | 8.7713 | 0.579 |
| TCGA-TN-A7HI-01 | OSCC | 13.5702 | 7.0992 | 11.4617 | 0.9409 |
| TCGA-CR-7389-01 | OSCC | 10.8941 | 4.2163 | 9.4037 | 3.2919 |
| TCGA-CN-6023-01 | OSCC | 9.2907 | 2.8304 | 9.7326 | 2.5803 |
| TCGA-CV-7430-01 | OSCC | 9.102 | 2.4509 | 8.3432 | 2.7002 |
| TCGA-HD-7832-01 | OSCC | 13.2398 | 2.6465 | 9.3238 | 2.381 |
| TCGA-T2-A6X0-01 | OSCC | 10.4905 | 3.6918 | 12.0978 | 0.7027 |
| TCGA-BB-4228-01 | OSCC | 10.5821 | 3.8018 | 9.6581 | 3.7606 |
| TCGA-HD-A633-01 | OSCC | 10.4584 | 4.7938 | 10.7422 | 3.6974 |
| TCGA-F7-A623-01 | OSCC | 15.135 | 5.0628 | 11.2698 | 3.7179 |
| TCGA-QK-A8ZA-01 | OSCC | 8.3774 | 3.2307 | 10.7784 | 3.9819 |
| TCGA-D6-8569-01 | OSCC | 12.0463 | 4.9548 | 9.915 | 3.4065 |
| TCGA-CV-6943-01 | OSCC | 11.3958 | 4.7865 | 9.0598 | 2.0589 |
| TCGA-CR-7367-01 | OSCC | 15.0799 | 6.2739 | 11.1947 | 3.4331 |
| TCGA-IQ-A61H-01 | OSCC | 13.0168 | 6.362 | 12.5619 | 3.0914 |
| TCGA-CN-A49C-01 | OSCC | 7.0902 | 2.3502 | 11.051 | 4.0824 |
| TCGA-CV-5441-01 | OSCC | 16.6407 | 4.4948 | 11.4148 | 0 |
| TCGA-WA-A7GZ-01 | OSCC | 15.4768 | 5.5875 | 10.391 | 4.4419 |
| TCGA-CN-A6V1-01 | OSCC | 9.5335 | 5.0129 | 11.3571 | 3.3256 |
| TCGA-CV-5971-01 | OSCC | 17.1218 | 5.4823 | 10.6446 | 1.2897 |
| TCGA-HD-A634-01 | OSCC | 13.7838 | 4.0343 | 10.1268 | 0.5972 |
| TCGA-TN-A7HJ-01 | OSCC | 14.4561 | 3.8264 | 12.1539 | 2.9247 |
| TCGA-CV-A464-01 | OSCC | 14.1841 | 7.6696 | 10.8227 | 2.238 |
| TCGA-CV-5970-01 | OSCC | 12.5191 | 4.366 | 10.5584 | 2.5923 |
| TCGA-BB-7862-01 | OSCC | 10.5189 | 5.2488 | 10.9902 | 2.5274 |
| TCGA-CN-5366-01 | OSCC | 11.4504 | 5.7978 | 9.8965 | 0.438 |
| TCGA-CV-5431-01 | OSCC | 11.5789 | 6.5992 | 10.3285 | 5.0436 |
| TCGA-CV-A6JM-01 | OSCC | 11.2196 | 7.617 | 9.8005 | 2.362 |
| TCGA-CR-5247-01 | OSCC | 15.4247 | 3.7072 | 8.6171 | 0 |
| TCGA-D6-A6EN-01 | OSCC | 11.7023 | 5.0601 | 10.4061 | 4.4698 |
| TCGA-P3-A6T8-01 | OSCC | 13.7754 | 7.2158 | 10.5694 | 3.8004 |
| TCGA-F7-A61V-01 | OSCC | 13.1829 | 5.6696 | 11.5066 | 0 |
| TCGA-BA-6873-01 | OSCC | 12.6236 | 3.6415 | 9.4206 | 3.1132 |
| TCGA-UF-A7JK-01 | OSCC | 11.0498 | 6.8794 | 10.5506 | 3.9498 |
| TCGA-H7-8501-01 | OSCC | 10.103 | 4.8113 | 9.963 | 3.3848 |
| TCGA-CN-5369-01 | OSCC | 12.5297 | 6.0503 | 10.903 | 2.2527 |
| TCGA-CN-5356-01 | OSCC | 14.0653 | 4.7044 | 9.609 | 0.709 |
| TCGA-CV-7407-01 | OSCC | 10.6397 | 3.8025 | 10.6432 | 3.1925 |
| TCGA-H7-7774-01 | OSCC | 11.9999 | 0.9313 | 9.6847 | 1.7082 |
| TCGA-QK-A6IF-01 | OSCC | 11.8286 | 4.9793 | 11.3985 | 10.0026 |
| TCGA-MT-A67A-01 | OSCC | 11.1942 | 6.8709 | 11.9903 | 3.0114 |
| TCGA-CX-A4AQ-01 | OSCC | 10.1319 | 7.5078 | 12.0804 | 7.9837 |
| TCGA-BB-8601-01 | OSCC | 9.3625 | 2.2839 | 9.7558 | 0.7181 |
| TCGA-QK-A6V9-01 | OSCC | 13.2177 | 2.8063 | 12.2692 | 2.3565 |
| TCGA-CR-6493-01 | OSCC | 17.0961 | 1.1072 | 9.422 | 1.7262 |
| TCGA-CR-7368-01 | OSCC | 15.2729 | 3.7077 | 11.3631 | 1.8673 |
| TCGA-CQ-7065-01 | OSCC | 12.1598 | 3.4654 | 10.4034 | 2.8861 |
| TCGA-F7-A624-01 | OSCC | 14.033 | 9.7994 | 10.417 | 0 |
| TCGA-CQ-5334-01 | OSCC | 12.1229 | 4.6741 | 8.7209 | 1.7479 |
| TCGA-CV-6939-01 | OSCC | 13.3971 | 5.4875 | 11.1637 | 4.0445 |
| TCGA-CV-5442-01 | OSCC | 12.3892 | 0.6077 | 10.9353 | 1.8557 |
| TCGA-BB-4227-01 | OSCC | 12.9517 | 4.3078 | 11.5584 | 2.1584 |
| TCGA-CV-7104-01 | OSCC | 14.6669 | 3.2481 | 9.7145 | 2.1952 |
| TCGA-CQ-7063-01 | OSCC | 10.0312 | 1.4544 | 10.8158 | 3.1893 |
| TCGA-CR-5249-01 | OSCC | 9.1497 | 2.9442 | 10.6508 | 1.5161 |
| TCGA-QK-A6II-01 | OSCC | 10.8372 | 5.1713 | 11.0422 | 2.6527 |
| TCGA-UF-A71D-01 | OSCC | 13.2467 | 3.3873 | 10.3852 | 0 |
| TCGA-CQ-A4CH-01 | OSCC | 10.8654 | 6.6778 | 10.5704 | 3.4239 |
| TCGA-CN-4741-01 | OSCC | 12.4313 | 4.9862 | 11.554 | 3.3027 |
| TCGA-IQ-A61J-01 | OSCC | 13.6815 | 6.3306 | 12.4199 | 1.2548 |
| TCGA-CV-7097-01 | OSCC | 14.5486 | 4.1399 | 8.9666 | 0.4625 |
| TCGA-CQ-6218-01 | OSCC | 12.2466 | 2.7008 | 8.9612 | 2.2656 |
| TCGA-CV-6952-01 | OSCC | 12.3871 | 2.9678 | 10.8537 | 2.2744 |
| TCGA-CV-A6JO-01 | OSCC | 8.8388 | 5.0563 | 11.6982 | 3.7663 |
| TCGA-CV-6954-01 | OSCC | 17.6322 | 3.295 | 9.5786 | 1.8924 |
| TCGA-CN-6013-01 | OSCC | 10.5088 | 4.5187 | 11.8145 | 3.6344 |
| TCGA-D6-6515-01 | OSCC | 12.6026 | 4.0104 | 8.9008 | 3.6479 |
| TCGA-BA-A6DD-01 | OSCC | 10.595 | 5.1478 | 9.6131 | 3.6221 |
| TCGA-CV-6959-01 | OSCC | 17.7049 | 4.2617 | 9.8251 | 0 |
| TCGA-QK-A8Z9-01 | OSCC | 12.6328 | 8.0802 | 11.3402 | 3.5856 |
| TCGA-CV-7414-01 | OSCC | 16.5495 | 2.8337 | 8.1282 | 5.7644 |
| TCGA-CQ-6224-01 | OSCC | 15.0919 | 4.9507 | 9.7674 | 1.7458 |
| TCGA-UF-A7JD-01 | OSCC | 15.1366 | 7.7945 | 12.0309 | 5.0485 |
| TCGA-BB-8596-01 | OSCC | 12.9762 | 8.3312 | 10.9928 | 1.5723 |
| TCGA-CV-7178-01 | OSCC | 13.153 | 5.2392 | 11.9807 | 2.9921 |
| TCGA-HD-A6HZ-01 | OSCC | 13.4335 | 8.5778 | 10.6566 | 3.9495 |
| TCGA-DQ-7596-01 | OSCC | 13.0989 | 0.677 | 9.9551 | 0.3778 |
| TCGA-KU-A66S-01 | OSCC | 14.9525 | 9.3332 | 11.061 | 7.8206 |
| TCGA-CV-7102-01 | OSCC | 13.8407 | 5.9788 | 9.4246 | 4.2494 |
| TCGA-BA-6870-01 | OSCC | 10.0485 | 3.554 | 11.365 | 3.2392 |
| TCGA-CV-7250-01 | OSCC | 15.3098 | 2.5749 | 10.1528 | 0.5368 |
| TCGA-CV-5439-01 | OSCC | 10.8952 | 3.9763 | 10.095 | 2.9074 |
| TCGA-CV-7440-01 | OSCC | 17.9248 | 2.1658 | 10.0246 | 1.8922 |
| TCGA-UF-A7J9-01 | OSCC | 12.154 | 7.8486 | 12.0779 | 3.6882 |
| TCGA-CQ-6223-01 | OSCC | 7.7919 | 2.7587 | 11.2138 | 3.6059 |
| TCGA-CR-7394-01 | OSCC | 10.5877 | 2.8969 | 9.8056 | 1.8397 |
| TCGA-CV-7103-01 | OSCC | 13.8937 | 4.3385 | 8.7207 | 0 |
| TCGA-P3-A5Q5-01 | OSCC | 9.4634 | 4.6249 | 11.0835 | 4.3735 |
| TCGA-CN-6019-01 | OSCC | 13.2629 | 6.693 | 8.9538 | 1.0287 |
| TCGA-CR-7380-01 | OSCC | 10.4722 | 4.8758 | 9.1368 | 2.5791 |
| TCGA-T2-A6WX-01 | OSCC | 10.8601 | 4.8854 | 11.5871 | 5.5035 |
| TCGA-CV-7425-01 | OSCC | 12.1264 | 4.4207 | 9.983 | 2.2626 |
| TCGA-CV-7446-01 | OSCC | 11.305 | 4.6787 | 13.3337 | 3.221 |
| TCGA-CN-5355-01 | OSCC | 16.2298 | 7.51 | 9.8958 | 2.18 |
| TCGA-D6-6826-01 | OSCC | 15.1768 | 5.9865 | 11.1336 | 2.1424 |
| TCGA-BA-5149-01 | OSCC | 11.2375 | 4.4285 | 9.7373 | 2.3524 |
| TCGA-CQ-5324-01 | OSCC | 11.0712 | 2.0937 | 8.1306 | 1.0632 |
| TCGA-CQ-7071-01 | OSCC | 10.9934 | 3.6547 | 11.3437 | 3.6547 |
| TCGA-CV-7423-01 | OSCC | 11.0288 | 2.881 | 10.5757 | 2.3908 |
| TCGA-HD-A6I0-01 | OSCC | 13.2922 | 7.3394 | 10.2673 | 2.1757 |
| TCGA-CV-6934-01 | OSCC | 13.9354 | 5.9891 | 9.8138 | 1.6839 |
| TCGA-CR-6491-01 | OSCC | 9.3751 | 2.2903 | 10.9857 | 3.4234 |
| TCGA-CR-7364-01 | OSCC | 16.353 | 1.4347 | 8.7006 | 0 |
| TCGA-P3-A6T0-01 | OSCC | 9.0635 | 5.9989 | 10.3612 | 3.5075 |
| TCGA-BA-5557-01 | OSCC | 11.7275 | 3.6219 | 9.0506 | 1.9755 |
| TCGA-UF-A7JO-01 | OSCC | 9.4595 | 5.3005 | 11.1514 | 2.5364 |
| TCGA-CV-6003-01 | OSCC | 10.6312 | 2.7193 | 10.4958 | 3.5075 |
| TCGA-CV-A45O-01 | OSCC | 8.8269 | 6.5082 | 13.6898 | 1.3222 |
| TCGA-CR-5250-01 | OSCC | 9.3162 | 2.6953 | 9.7208 | 4.5422 |
| TCGA-CV-7095-01 | OSCC | 12.9044 | 3.3875 | 9.7436 | 1.0367 |
| TCGA-CQ-A4CG-01 | OSCC | 9.6186 | 5.8865 | 12.3551 | 4.1341 |
| TCGA-CQ-5333-01 | OSCC | 15.2655 | 5.8548 | 10.2699 | 5.1393 |
| TCGA-HD-7917-01 | OSCC | 17.1238 | 6.5677 | 11.5319 | 2.381 |
| TCGA-CV-6935-01 | OSCC | 10.6492 | 3.1917 | 9.4159 | 4.9779 |
| TCGA-CN-A63U-01 | OSCC | 12.8909 | 4.7692 | 12.4362 | 0 |
| TCGA-UF-A719-01 | OSCC | 15.8678 | 2.3422 | 10.5418 | 1.4309 |
| TCGA-CV-7243-01 | OSCC | 13.5863 | 4.9921 | 9.466 | 4.7126 |
| TCGA-CV-6933-01 | OSCC | 11.2501 | 5.3479 | 10.9279 | 2.7486 |
| TCGA-CV-7248-01 | OSCC | 11.1441 | 5.2341 | 9.9966 | 3.356 |
| TCGA-CV-A6JY-01 | OSCC | 15.163 | 3.7615 | 12.4334 | 4.2366 |
| TCGA-QK-A652-01 | OSCC | 14.6029 | 7.0668 | 11.2525 | 3.2876 |
| TCGA-CN-A6UY-01 | OSCC | 8.6796 | 2.8205 | 10.7994 | 1.6804 |
| TCGA-MT-A51W-01 | OSCC | 8.5521 | 3.9502 | 11.0182 | 3.566 |
| TCGA-CQ-6229-01 | OSCC | 14.2978 | 4.5523 | 11.0705 | 1.4478 |
| TCGA-UF-A718-01 | OSCC | 8.8282 | 5.5373 | 11.3544 | 1.6617 |
| TCGA-QK-A64Z-01 | OSCC | 9.2073 | 7.2105 | 10.7707 | 4.7502 |
| TCGA-CN-4731-01 | OSCC | 17.6236 | 4.6752 | 10.9102 | 3.5426 |
| TCGA-CV-7418-01 | OSCC | 12.1152 | 4.6129 | 10.7462 | 4.5407 |
| TCGA-IQ-7631-01 | OSCC | 13.5434 | 7.4421 | 11.7317 | 0 |
| TCGA-D6-6516-01 | OSCC | 9.6071 | 1.62 | 9.2793 | 1.62 |
| TCGA-CV-5436-01 | OSCC | 13.9463 | 7.8507 | 10.1942 | 3.1802 |
| TCGA-QK-A6IH-01 | OSCC | 15.6173 | 5.5732 | 11.265 | 3.8397 |
| TCGA-CV-7238-01 | OSCC | 14.6826 | 7.0798 | 12.4222 | 4.6634 |
| TCGA-P3-A6SW-01 | OSCC | 9.7634 | 6.1261 | 11.4845 | 4.8968 |
| TCGA-CR-6467-01 | OSCC | 9.2655 | 3.5353 | 11.604 | 3.9356 |
| TCGA-CN-6011-01 | OSCC | 11.8793 | 3.8128 | 10.2979 | 3.6265 |
| TCGA-CN-6997-01 | OSCC | 10.6576 | 5.7381 | 11.7685 | 3.9291 |
| TCGA-BB-7866-01 | OSCC | 14.6169 | 4.004 | 10.8343 | 2.2513 |
| TCGA-CR-7386-01 | OSCC | 12.2744 | 4.6806 | 9.18 | 3.5004 |
| TCGA-BB-A6UM-01 | OSCC | 9.6071 | 3.3484 | 11.1031 | 0 |
| TCGA-BA-5555-01 | OSCC | 8.1824 | 1.5551 | 8.6555 | 0 |
| TCGA-IQ-A6SG-01 | OSCC | 11.4422 | 5.3304 | 11.1629 | 1.642 |
| TCGA-CV-6956-01 | OSCC | 16.6378 | 4.7704 | 10.674 | 5.1842 |
| TCGA-CV-5435-01 | OSCC | 12.6161 | 3.642 | 10.0176 | 3.139 |
| TCGA-CN-A49A-01 | OSCC | 16.3115 | 9.0037 | 12.7567 | 6.3574 |
| TCGA-HD-8314-01 | OSCC | 13.6854 | 8.16 | 10.6662 | 0.6162 |
| TCGA-P3-A5QE-01 | OSCC | 8.0573 | 2.4572 | 10.8232 | 3.2643 |
| TCGA-D6-6824-01 | OSCC | 12.624 | 4.3657 | 9.7477 | 1.2638 |
| TCGA-BA-5151-01 | OSCC | 9.8916 | 5.0685 | 10.392 | 2.0711 |
| TCGA-CV-7438-01 | OSCC | 14.1758 | 4.9302 | 10.3287 | 4.524 |
| TCGA-T2-A6WZ-01 | OSCC | 11.4105 | 7.1507 | 11.3437 | 3.6086 |
| TCGA-CN-5361-01 | OSCC | 11.8377 | 4.9534 | 8.694 | 3.1597 |
| TCGA-CV-7090-01 | OSCC | 10.4224 | 4.5902 | 9.3465 | 2.9 |
| TCGA-P3-A6T2-01 | OSCC | 13.4465 | 5.8854 | 10.2939 | 1.6565 |
| TCGA-CR-7372-01 | OSCC | 12.1365 | 4.0856 | 9.053 | 1.8444 |
| TCGA-CV-7261-01 | OSCC | 10.4935 | 4.9904 | 9.5737 | 2.314 |
| TCGA-CN-A6V6-01 | OSCC | 12.725 | 4.6593 | 11.1285 | 2.2726 |
| TCGA-CV-7235-01 | OSCC | 11.7851 | 3.3951 | 10.2723 | 2.9635 |
| TCGA-CN-6995-01 | OSCC | 13.2865 | 2.3629 | 10.205 | 3.2784 |
| TCGA-BA-A6DL-01 | OSCC | 16.8798 | 5.4172 | 11.4705 | 0 |
| TCGA-QK-AA3J-01 | OSCC | 15.8979 | 5.0802 | 10.1781 | 6.9247 |
| TCGA-IQ-A61O-01 | OSCC | 18.2458 | 6.7135 | 11.1701 | 6.2523 |
| TCGA-CV-6938-01 | OSCC | 8.612 | 3.8489 | 10.2712 | 2.7325 |
| TCGA-CQ-6219-01 | OSCC | 12.1702 | 4.1921 | 9.0413 | 2.4614 |
| TCGA-CN-6996-01 | OSCC | 14.0102 | 5.9441 | 9.915 | 2.9001 |
| TCGA-TN-A7HL-01 | OSCC | 9.7316 | 4.5509 | 10.2686 | 0 |
| TCGA-BB-4223-01 | OSCC | 12.7568 | 3.8195 | 11.3863 | 2.8185 |
| TCGA-CQ-5331-01 | OSCC | 12.3833 | 3.2444 | 9.9313 | 2.0385 |
| TCGA-BA-A8YP-01 | OSCC | 12.2314 | 6.0907 | 10.6933 | 3.7673 |
| TCGA-CV-7242-01 | OSCC | 15.3179 | 3.6148 | 10.5323 | 5.416 |
| TCGA-CN-5373-01 | OSCC | 11.1367 | 3.1148 | 10.9285 | 3.8962 |
| TCGA-CN-5370-01 | OSCC | 13.5735 | 5.1116 | 9.4567 | 1.6228 |
| TCGA-DQ-7593-01 | OSCC | 12.7233 | 3.3647 | 11.0661 | 1.315 |
| TCGA-CV-5432-01 | OSCC | 12.0792 | 6.4858 | 9.861 | 2.2544 |
| TCGA-CV-7428-01 | OSCC | 18.7341 | 3.5612 | 10.6477 | 5.045 |
| TCGA-F7-A620-01 | OSCC | 12.6428 | 5.3487 | 10.9165 | 1.9798 |
| TCGA-CN-5364-01 | OSCC | 12.9341 | 5.119 | 9.8324 | 1.5236 |
| TCGA-UF-A7JH-01 | OSCC | 16.7512 | 7.3458 | 11.6637 | 1.0121 |
| TCGA-HL-7533-01 | OSCC | 9.2094 | 3.6323 | 9.94 | 5.5976 |
| TCGA-F7-A50G-01 | OSCC | 12.9526 | 6.2513 | 11.7722 | 3.2993 |
| TCGA-CN-A498-01 | OSCC | 11.0253 | 3.6886 | 11.1279 | 4.02 |
| TCGA-CV-6962-01 | OSCC | 13.1422 | 4.5491 | 10.8544 | 2.2647 |
| TCGA-BA-A4IH-01 | OSCC | 10.756 | 4.2361 | 10.4167 | 5.1068 |
| TCGA-P3-A6SX-01 | OSCC | 13.1113 | 11.2677 | 11.6483 | 5.1082 |
| TCGA-CN-4726-01 | OSCC | 10.4131 | 4.8782 | 10.9174 | 1.9631 |
| TCGA-CR-7393-01 | OSCC | 11.4625 | 4.0925 | 9.6108 | 1.5125 |
| TCGA-CQ-7067-01 | OSCC | 13.085 | 6.716 | 10.5183 | 3.7544 |
| TCGA-BA-4076-01 | OSCC | 7.9428 | 6.9578 | 11.1381 | 4.314 |
| TCGA-CR-6471-01 | OSCC | 9.6576 | 2.6935 | 9.9224 | 2.0444 |
| TCGA-CN-A499-01 | OSCC | 14.6821 | 3.8145 | 11.2044 | 3.2375 |
| TCGA-QK-A6VC-01 | OSCC | 9.9302 | 7.1039 | 12.4864 | 3.897 |
| TCGA-DQ-5629-01 | OSCC | 9.0622 | 3.6868 | 8.7703 | 1.7551 |
| TCGA-MZ-A5BI-01 | OSCC | 10.1462 | 4.4579 | 10.5549 | 5.5949 |
| TCGA-D6-A6EO-01 | OSCC | 14.8968 | 3.4763 | 10.2119 | 0 |
| TCGA-MZ-A7D7-01 | OSCC | 6.8658 | 3.5028 | 11.1419 | 2.2857 |
| TCGA-H7-A6C4-01 | OSCC | 15.5924 | 6.6149 | 10.6992 | 2.0171 |
| TCGA-QK-A6IJ-01 | OSCC | 15.8075 | 6.9259 | 11.4036 | 3.4427 |
| TCGA-CV-7424-01 | OSCC | 11.2815 | 6.5076 | 11.327 | 4.0204 |
| TCGA-CV-A45Y-01 | OSCC | 10.7149 | 7.4664 | 11.163 | 2.9289 |
| TCGA-CV-5434-01 | OSCC | 12.8338 | 5.8968 | 9.9324 | 3.0571 |
| TCGA-CR-7397-01 | OSCC | 12.5952 | 5.6708 | 10.7247 | 2.7389 |
| TCGA-CR-6474-01 | OSCC | 13.0294 | 5.4099 | 9.5659 | 2.2093 |
| TCGA-WA-A7H4-01 | OSCC | 14.5209 | 9.5263 | 11.1066 | 3.4081 |
| TCGA-CN-A63W-01 | OSCC | 18.173 | 4.5633 | 10.8673 | 2.2343 |
| TCGA-HD-7229-01 | OSCC | 10.1581 | 2.9398 | 10.5765 | 2.7621 |
| TCGA-CV-6951-01 | OSCC | 15.8738 | 3.3804 | 11.6091 | 2.8911 |
| TCGA-CV-A6JD-01 | OSCC | 12.1128 | 6.87 | 10.3902 | 5.4558 |
| TCGA-CQ-6220-01 | OSCC | 10.3375 | 6.6336 | 10.5224 | 1.6547 |
| TCGA-CV-A465-01 | OSCC | 11.8939 | 5.5194 | 10.6912 | 3.288 |
| TCGA-CQ-A4CD-01 | OSCC | 9.4228 | 5.8967 | 11.5905 | 4.2974 |
| TCGA-KU-A6H8-01 | OSCC | 17.9231 | 9.584 | 11.4887 | 1.5308 |
| TCGA-CN-5360-01 | OSCC | 12.5685 | 5.6539 | 10.356 | 1.6534 |
| TCGA-UF-A7JJ-01 | OSCC | 12.2907 | 8.3742 | 11.9915 | 3.2452 |
| TCGA-CN-A497-01 | OSCC | 14.0584 | 5.4067 | 11.5356 | 0 |
| TCGA-D6-A4Z9-01 | OSCC | 13.078 | 5.0517 | 9.7117 | 1.2745 |
| TCGA-UF-A7JA-01 | OSCC | 12.6847 | 7.5825 | 11.0276 | 5.4263 |
| TCGA-CQ-5329-01 | OSCC | 12.0715 | 5.4864 | 7.4357 | 2.6806 |
| TCGA-CV-7409-01 | OSCC | 16.9022 | 3.7153 | 10.3159 | 2.2607 |
| TCGA-CR-7399-01 | OSCC | 7.2465 | 3.2859 | 9.5 | 3.0522 |
| TCGA-CV-6961-01 | OSCC | 15.6982 | 3.6217 | 9.5693 | 2.7026 |
| TCGA-CQ-6225-01 | OSCC | 17.1897 | 3.7231 | 11.2428 | 2.5912 |
| TCGA-F7-A50I-01 | OSCC | 8.8483 | 6.701 | 12.3737 | 2.5462 |
| TCGA-QK-A6VB-01 | OSCC | 17.8741 | 7.5414 | 10.9337 | 3.2314 |
| TCGA-CV-7261-11 | Normal | 18.2665 | 3.0902 | 9.9922 | 0 |
| TCGA-CV-7245-11 | Normal | 16.3197 | 8.4098 | 10.7984 | 0 |
| TCGA-CV-7434-11 | Normal | 11.1169 | 7.1072 | 10.1634 | 0 |
| TCGA-CV-7406-11 | Normal | 17.1797 | 9.3691 | 10.8301 | 0.6447 |
| TCGA-CV-7424-11 | Normal | 15.7692 | 5.5122 | 11.2785 | 0 |
| TCGA-CV-6943-11 | Normal | 12.3326 | 3.7839 | 10.1354 | 0 |
| TCGA-HD-8635-11 | Normal | 13.5854 | 8.0972 | 10.4778 | 0 |
| TCGA-CV-6934-11 | Normal | 14.4363 | 3.7804 | 9.2567 | 2.6916 |
| TCGA-CV-6935-11 | Normal | 15.6582 | 1.4268 | 9.002 | 0.883 |
| TCGA-CV-6956-11 | Normal | 14.7295 | 6.6021 | 10.8155 | 0 |
| TCGA-CV-7178-11 | Normal | 8.785 | 5.6191 | 11.558 | 2.4117 |
| TCGA-CV-7242-11 | Normal | 17.6438 | 4.4755 | 10.0402 | 0 |
| TCGA-CV-7432-11 | Normal | 8.4254 | 6.4944 | 9.066 | 0 |
| TCGA-CV-7425-11 | Normal | 14.5698 | 2.0247 | 9.9787 | 2.8355 |
| TCGA-CV-7437-11 | Normal | 14.5865 | 2.7516 | 9.7531 | 0 |
| TCGA-CV-7183-11 | Normal | 15.573 | 2.0017 | 9.0347 | 0 |
| TCGA-CV-7423-11 | Normal | 14.3779 | 4.3608 | 10.599 | 0.9835 |
| TCGA-CV-7177-11 | Normal | 15.7806 | 3.9979 | 10.5319 | 2.1137 |
| TCGA-CV-7235-11 | Normal | 10.6306 | 5.4102 | 9.4725 | 0 |
| TCGA-CV-6959-11 | Normal | 14.6632 | 5.7496 | 10.9131 | 0 |
| TCGA-CV-7250-11 | Normal | 15.0207 | 4.8922 | 10.0125 | 0 |
| TCGA-CV-7097-11 | Normal | 16.9612 | 8.4996 | 10.2912 | 0 |
| TCGA-CV-7103-11 | Normal | 13.2034 | 4.9892 | 10.0142 | 0 |
| TCGA-H7-A6C4-11 | Normal | 15.6505 | 8.6859 | 11.8489 | 0 |
| TCGA-CV-6960-11 | Normal | 10.8122 | 3.6216 | 9.636 | 0 |
| TCGA-CV-6939-11 | Normal | 17.718 | 9.7107 | 10.5522 | 0 |
| TCGA-CV-6955-11 | Normal | 9.1956 | 4.1245 | 10.0644 | 1.6113 |
| TCGA-CV-7238-11 | Normal | 15.0738 | 6.9115 | 9.7115 | 0 |
| TCGA-WA-A7GZ-11 | Normal | 17.0972 | 10.9762 | 11.4902 | 0.7153 |
| TCGA-CV-6961-11 | Normal | 13.2759 | 3.5069 | 9.688 | 0 |
| TCGA-CV-6938-11 | Normal | 9.1212 | 5.0484 | 11.1292 | 2.6207 |
| TCGA-CV-6933-11 | Normal | 10.3126 | 6.0502 | 9.7113 | 1.8116 |
| TCGA-CV-7255-11 | Normal | 11.7489 | 4.6645 | 10.4456 | 0 |
| TCGA-HD-A6I0-11 | Normal | 15.9948 | 8.764 | 11.6004 | 1.3453 |
| TCGA-CV-7101-11 | Normal | 14.7028 | 3.1896 | 9.2042 | 0 |
| TCGA-CV-6936-11 | Normal | 15.0288 | 4.4701 | 10.1792 | 0 |
| TCGA-CV-7252-11 | Normal | 12.7746 | 6.3543 | 11.6729 | 0 |
| TCGA-CV-7438-11 | Normal | 12.1833 | 1.2425 | 9.3858 | 2.2151 |
| TCGA-CV-7416-11 | Normal | 12.2123 | 5.4653 | 11.171 | 0 |
| TCGA-CV-6962-11 | Normal | 17.462 | 4.5019 | 9.825 | 0 |
| TCGA-CV-7440-11 | Normal | 16.5702 | 3.9359 | 8.6359 | 0 |
| TCGA-CV-7091-11 | Normal | 17.7183 | 7.8326 | 10.2483 | 0 |
| TCGA-HD-A6HZ-11 | Normal | 14.5151 | 10.061 | 12.1862 | 0 |
| TCGA-H7-A6C5-11 | Normal | 14.5035 | 5.9366 | 10.1579 | 0 |
